# Heterostructures of ε-Fe_2_O_3_ and α-Fe_2_O_3_: insights from density functional theory

**DOI:** 10.1039/d0ra04020g

**Published:** 2020-07-22

**Authors:** Imran Ahamed, Nicola Seriani, Ralph Gebauer, Arti Kashyap

**Affiliations:** School of Basic Sciences, Indian Institute of Technology Mandi Himachal Pradesh 175005 India; The Abdus Salam International Centre for Theoretical Physics (ICTP) Strada Costiera 11 34151 Trieste Italy; School of Basic Sciences, School of Computing and Electrical Engineering, Indian Institute of Technology Mandi Himachal Pradesh 175005 India arti@iitmandi.ac.in

## Abstract

Many materials used in energy devices or applications suffer from the problem of electron–hole pair recombination. One promising way to overcome this problem is the use of heterostructures in place of a single material. If an electric dipole forms at the interface, such a structure can lead to a more efficient electron–hole pair separation and thus prevent recombination. Here we model and study a heterostructure comprised of two polymorphs of Fe_2_O_3_. Each one of the two polymorphs, α-Fe_2_O_3_ and ε-Fe_2_O_3_, individually shows promise for applications in photoelectrochemical cells. The heterostructure of these two materials is modeled by means of density functional theory. We consider both ferromagnetic as well as anti-ferromagnetic couplings at the interface between the two systems. Both individual oxides are insulating in nature and have an anti-ferromagnetic spin arrangement in their ground state. The same properties are found also in their heterostructure. The highest occupied electronic orbitals of the combined system are localized at the interface between the two iron-oxides. The localization of charges at the interface is characterized by electrons residing close to the oxygen atoms of ε-Fe_2_O_3_ and electron–holes localized on the iron atoms of α-Fe_2_O_3_, just around the interface. The band alignment at the interface of the two oxides shows a type-III broken band-gap heterostructure. The band edges of α-Fe_2_O_3_ are higher in energy than those of ε-Fe_2_O_3_. This band alignment favours a spontaneous transfer of excited photo-electrons from the conduction band of α- to the conduction band of ε-Fe_2_O_3_. Similarly, photo-generated holes are transferred from the valence band of ε- to the valence band of α-Fe_2_O_3_. Thus, the interface favours a spontaneous separation of electrons and holes in space. The conduction band of ε-Fe_2_O_3_, lying close to the valence band of α-Fe_2_O_3_, can result in band-to-band tunneling of electrons which is a characteristic property of such type-III broken band-gap heterostructures and has potential applications in tunnel field-effect transistors.

## Abstract 2

Electron–hole pair recombination is reduced in heterostructures if used in devices in place of single material.

## Graphical abstract

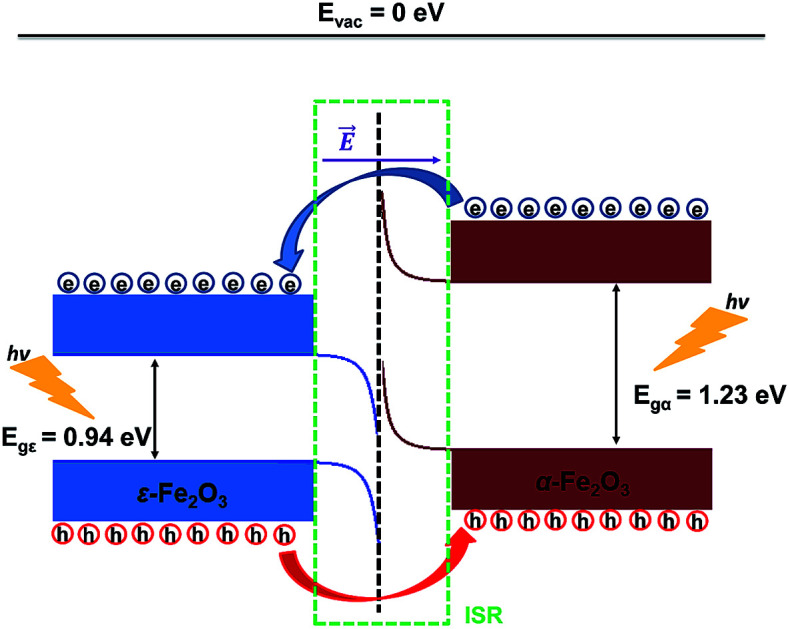

## Introduction

At the interface between two different materials one can often observe new emergent physical properties and phenomena which are not found in the individual materials.^1,2^ For example, LaAlO_3_ and SrTiO_3_ both are insulating materials, but in a heterostructure, the interface of these systems was found to be metallic.^3^ In general, oxides can have properties ranging from ferroelectric to piezoelectric, bandgap insulating or superconducting, *etc.* Such properties are related to the lattice structure and the symmetry of the materials. By forming heterostructures of these oxides, the crystal lattice is disturbed and the symmetries are broken, which alters the properties of the combined system. Using various techniques, heterostructures of oxides can be prepared with novel properties, such as the presence of two dimensional electron gas (2DEG) at the interface of LaAlO_3_/SrTiO_3_ (ref. 3) and also in KNbO_3_/BaTiO_3_, KNbO_3_/PbTiO_3_, KNbO_3_/SrTiO_3_ heterointerfaces.^4^ Very high electron mobilities ∼ 10^6^ cm^2^ V^−1^ s^−1^ were observed at the heterointerface of MgZnO/ZnO.^5^ The interface of LaAlO_3_/SrTiO_3_ was also found to be superconductive.^6,7^ Very recently, a high-mobility spin-polarized 2DEG was observed at the interface of EuO/KTaO_3_.^8^ Emergent giant topological Hall effect is also observed in heterostructures of LaSrMnO_3_/SrIrO_3_.^9^

Heterostructures can also be important for electron–hole separation in photoactive devices. Here we are interested in iron oxides that have demonstrated potential as photocatalysts, but suffer from high recombination. Bulk ε-Fe_2_O_3_ is an indirect band-gap semiconducting material with a gap of 1.9 eV,^10,11^ whereas bulk α-Fe_2_O_3_ is a direct band-gap semiconducting material with 2.2 eV of band-gap.^12,13^ Bulk ε-Fe_2_O_3_ is magnetically hard with a room temperature coercivity of 20 kOe,^14–16^ while bulk α-Fe_2_O_3_ is magnetically very soft with a room temperature coercivity of a few 100 Oe.^17–19^ Single crystals of ε-Fe_2_O_3_ are not naturally found nor prepared experimentally, but it is always obtained in mixtures with α-Fe_2_O_3_ and its other polymorphs. Also, both ε- and α-Fe_2_O_3_ being charge-transfer insulators,^10,20^ the heterostructures of these two polymorphs can show exciting phenomena at the interface, just like various other transition metals oxide heterostructures.

Both these phases of iron-oxide have been theoretically studied and also experimentally proven for H_2_ production from sunlight in photoelectrochemical (PEC) cells with different production rates.^21–28^ The application of ε- and α-Fe_2_O_3_ in energy devices such as PEC cells suffers from the presence of surface states acting as trap sites for electron–holes which also favour the recombination of photo-generated electron–hole pairs.^27,29^ To increase the efficiency of the PEC cells with α-Fe_2_O_3_ photoelectrodes, the surface states can be passivated by growing overlayers of Al_2_O_3_, Ga_2_O_3_ or In_2_O_3_.^30–32^ The efficiency of ε-Fe_2_O_3_ is found much higher in H_2_ production in PEC cells in comparison with α-Fe_2_O_3_.^28^ Like in the case of BiFeO_3_,^33,34^ the magnetoelectric/ferroelectric nature of ε-Fe_2_O_3_ (ref. 35) reduces the recombination of photo-generated charges thus giving higher H_2_ yield in comparison with α-Fe_2_O_3_ used in PEC cells.

Heterostructures are proven to show a great amount of reduction of electron–hole recombination by separating the two charges.^36–38^ The energy band alignment of the two materials at the interface of the heterostructure of BiFeO_3_/ε-Fe_2_O_3_ is such that it facilitates the separation of electron–hole pairs.^39^ Very recently, epitaxial thin films of α-Fe_2_O_3_ was grown on multiferroic ε-Fe_2_O_3_ supported on SrTiO_3_ as a substrate for a possible application as a 4-resistive state multiferroic tunnel junction (MFTJ).^40^ Since then, heterojunctions of semiconductors, insulators or semiconductor–insulator junctions show unique electronic and magnetic properties. For this reason, we have explored the heterostructure of two semiconducting oxides, namely the two different polymorphs of Fe_2_O_3_.

Here, we have investigated the heterostructures of Fe_2_O_3_ by first-principles calculations. We modelled the heterointerface of ε-Fe_2_O_3_ and α-Fe_2_O_3_, the two polymorphs of Fe_2_O_3_. The interface formation energy of the heterointerface is calculated for the various magnetic couplings, yielding the stable magnetic ordering in the heterostructure. The electronic structure of the heterostructure of the anti-ferromagnetic α-Fe_2_O_3_ and multiferroic ε-Fe_2_O_3_ is calculated and the interface states are determined. We have obtained the charge transfer in the ε/α-Fe_2_O_3_ system by means of the charge density difference and also from the band alignment. We have shown the band alignment at the interface of ε-Fe_2_O_3_ and α-Fe_2_O_3_ subsystems forming the heterostructure by taking a common vacuum level as reference for the combined system as well as for the individual subsystems. In this way, the band offset and the direction of the charge flow across the interface is determined.

## Calculation details

We have performed spin-polarized density functional theory (DFT) calculations as implemented in VASP (Vienna *Ab initio* Simulation Package).^41–43^ The Perdew–Burke–Ernzerhoff (PBE)^44^ form of the generalized gradient approximation was used for the treatment of the exchange–correlation effects. We have used the projected augmented wave (PAW)^45^ method and pseudo-potentials with d^7^s^1^ and s^2^p^4^ as the valence configurations for Fe- and O-atoms, respectively. The DFT+*U* formalism^46,47^ was used to account for the strongly correlated nature of the localized electrons. An effective Hubbard-*U* parameter^48^ is introduced. This *U*-correction is applied to the Fe 3d-states, and its value is chosen to be 4 eV. This value is commonly used for hematite.^20^*U* − *J* = 4 eV is reported to give a band gap in close agreement with the *ab initio* study for bulk ε-Fe_2_O_3_ (ref. 10 and 11) and also for bulk α-Fe_2_O_3_.^20^ The *U* value chosen for the Fe-atoms are same for the surface and bulk atoms as opposed to the work of Lewandowski *et al.*^49^ because the Fe-atoms at the interface has the same environment above and below it. Structural optimizations of each slab of Fe_2_O_3_ and of their heterostructure and calculation of the density of states of the heterostructure were carried out using a Monkhorst–Pack (M–P)^50^*k*-point mesh of 5 × 3 × 1 points in the Brillouin zone. To represent the electronic wave orbitals, we have used a plane-wave basis set with an energy cutoff of 530 eV. The atoms of the heterostructure were selectively relaxed in the *z*-direction only in a constant volume cell using a conjugate gradient optimization^51^ algorithm. The convergence criteria for electronic self-consistency was set to 10^−7^ eV and for the forces in relaxations to 0.005 eV Å^−1^ for each atom. Due to the non-centrosymmetric nature of bulk ε-Fe_2_O_3_ and also the heterostructures of ε/α-Fe_2_O_3_, the two surfaces are not same and thus are not dipole neutral. In order to apply a dipole correction, compensating dipoles^52,53^ are introduced in the vacuum region of the slab of each iron oxide and also for their heterostructure.

## Modelling of heterostructure

**Fig. 1:**
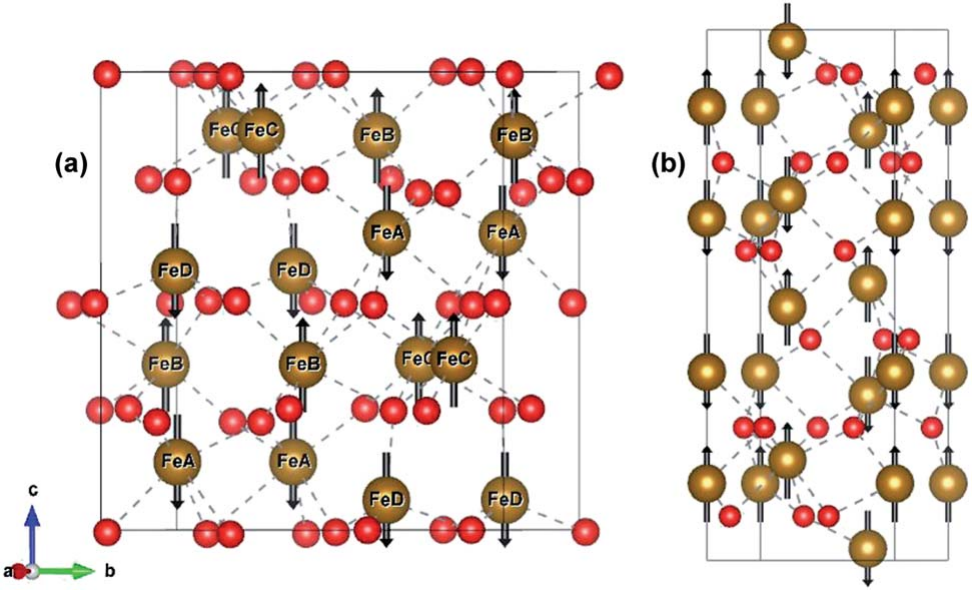
Bulk structure of (a) ε-Fe_2_O_3_ and (b) α-Fe_2_O_3_. Bonds are shown in broken grey lines. The arrows represents the direction of the magnetic moment of the Fe-atoms. Red and brown atoms represents the O- and Fe-atoms, respectively.

ε-Fe_2_O_3_ has an orthorhombic structure with space group *Pna*2_1_. The DFT-optimized lattice parameters were found to be *a* = 5.125 Å, *b* = 8.854 Å and *c* = 9.563 Å which are in good agreement with the experimental lattice parameters.^27^ The bulk unit cell contains eight formula units of Fe_2_O_3_ having four inequivalent Fe sites. The four inequivalent (Fe_A_, Fe_B_, Fe_C_, Fe_D_) type atoms have the following respective spins: *β*, *α*, *α*, *β*, which gives an A-type anti-ferromagnetic coupling as shown in Fig. 1(a) α-Fe_2_O_3_ has a corundum structure and there are six formula units of Fe_2_O_3_ in its unit cell. The structure of hematite is rhombohedrally centered hexagonal with space group *R*3̄*c* having DFT optimized lattice parameters as *a* = 5.038 Å and *c* = 13.772 Å which are in close agreement with the experimental lattice parameter.^54,55^ It consists of hexagonal closed pack arrays of oxygen stacked along the [001] direction. Hematite has an anti-ferromagnetic spin arrangement as shown in Fig. 1(b) and has net zero magnetization.

**Fig. 2:**
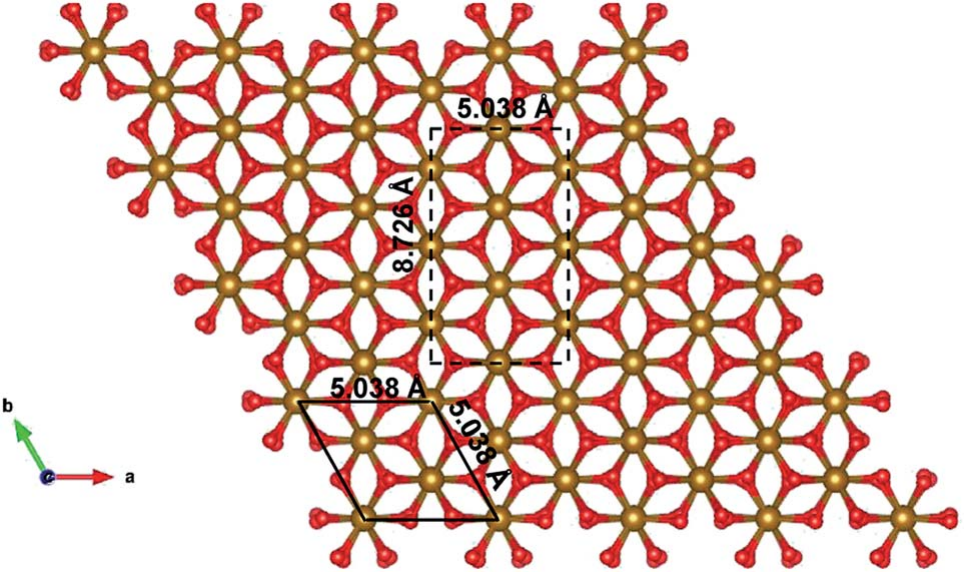
Modelled orthorhombic structure of α-Fe_2_O_3_ from the supercell of hexagonal unit cell of α-Fe_2_O_3_. Bulk unit cell is in black solid line and the modelled orthorhombic cell is in black broken line.

Since the unit cell of α-Fe_2_O_3_ and ε-Fe_2_O_3_ is hexagonal and orthorhombic, respectively, the lattice mismatch is huge and the modelling of the interface is difficult. For the interface modelling, we have taken one unit cell thick slab of ε-Fe_2_O_3_ having 8 formula units of Fe_2_O_3_ and an orthorhombic slab of α-Fe_2_O_3_ having one oxygen atom less than 11 formula units of Fe_2_O_3_ modelled from its hexagonal super cell as shown in Fig. 2.

**Fig. 3:**
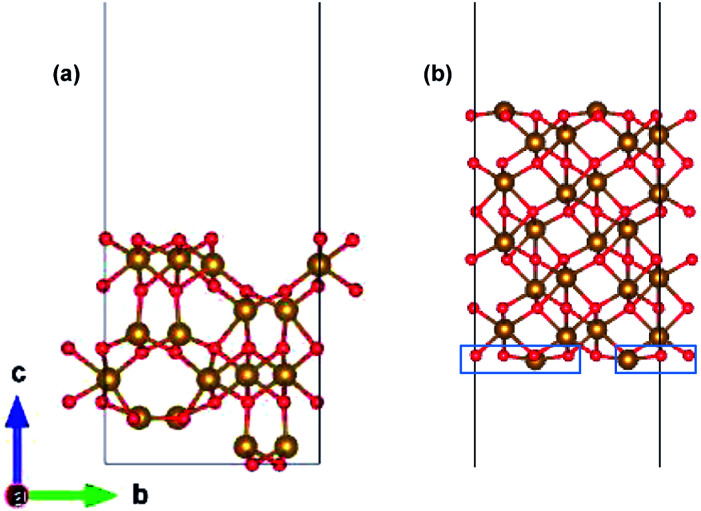
Optimized slab of (a) ε-Fe_2_O_3_ and (b) α-Fe_2_O_3_ for the preparation of the heterostructure.

The preferred growth direction of the slabs of ε-Fe_2_O_3_ and α-Fe_2_O_3_ is along the [001] direction.^40,50–61^ The slabs are prepared from optimized bulk structures by the supercell approach in the crystallographic *c*-axis with a vacuum of 15 Å and the ions were allowed to relax. The lattice mismatch between the orthorhombic ε-Fe_2_O_3_ and the modelled orthorhombic α-Fe_2_O_3_ is 1.69% and 1.44% along the *x*- and *y*-directions, respectively. The slab of ε-Fe_2_O_3_ and α-Fe_2_O_3_ have the same thickness as that of its bulk unit cell, *i.e.*, of 9.563 Å and 13.772 Å, respectively. The layers of α-Fe_2_O_3_ consist of Fe-atoms in octahedral coordination with oxygen, so the only choice would be to have an oxygen or an iron terminated surface. However, this cancels out with the choice of the termination in the other phase, because the interface must respect the alternation of iron and oxygen layers in order to be stable. For ε-Fe_2_O_3_, the layers consist of Fe-atoms in octahedral, tetrahedral, and a mix of octahedral and tetrahedral coordination with oxygen atoms. In order to make a perfect interface with the α-Fe_2_O_3_ so that we have an interface of low defect and low trap density, we have chosen the ε-Fe_2_O_3_ slab with a top surface as an octahedral coordination. Any other choice would greatly increase the number of interface atoms with non-optimal coordination. The optimized orthorhombic slabs of both iron-oxides for the heterostructure modelling are shown in Fig. 3.

The heterostructure is modelled by placing the slab of α-Fe_2_O_3_ on top of ε-Fe_2_O_3_ with a separation of 2 Å and allowed to selectively relax in the *z*-direction of the heterostructure with 15 Å vacuum provided along *z*-direction. Since both these phases of Fe_2_O_3_ have layered anti-ferromagnetic spin arrangement, therefore the interface could be prepared with ferromagnetic or anti-ferromagnetic coupling between ε- and α-Fe_2_O_3_ at the interface. The interface heterostructure of ε/α-Fe_2_O_3_ is obtained by removing the two Fe-atoms and four O-atoms (marked in blue rectangle) from the bottom of the slab of α-Fe_2_O_3_ and combining it with the ε-Fe_2_O_3_ slab. The 2 × FeO_2_ units are removed from the bottom surface of α-Fe_2_O_3_ in order to make a perfectly coordinated interface consisting of only octahedrally coordinated Fe-atoms.

## Results and discussion

### Interface stability

The stability of the modelled heterostructure is checked by calculating the interface formation energy of the heterostructure having different magnetic couplings at the interface. The interface formation energy (*E*_form_)^62^ is expressed as1
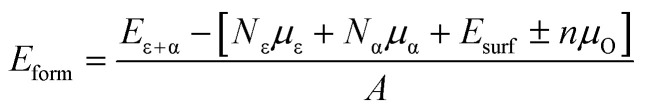
where *E*_ε+α_ is the DFT total energy of the ε- and α-Fe_2_O_3_ heterostructure; *N*_ε_ and *N*_α_ are the number of formula units of bulk ε-Fe_2_O_3_ and α-Fe_2_O_3_, respectively, in the heterostructure; *μ*_ε_ and *μ*_α_ are the chemical potential of bulk ε-Fe_2_O_3_ and α-Fe_2_O_3_ per formula unit, respectively; *E*_surf_ is the sum of the surface energy of the top and bottom surfaces of the heterostructure system; *n* is the number of O atoms in excess/deficient (+/−) relative to the ε-Fe_2_O_3_ and α-Fe_2_O_3_ stoichiometry; *μ*_O_ is the chemical potential of oxygen vapour and taken as half of the chemical potential of oxygen molecule and *A* is the interface area.

The surface energy (*E*_surf_) used in eqn (1) is obtained from the sum of two separate calculations for both the bottom and top surface made due to the slabs of ε-Fe_2_O_3_ and α-Fe_2_O_3_, respectively, by using the equation^25,27^ as2
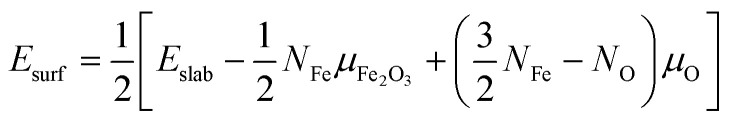
where *E*_slab_ is the total energy of the respective slab; *N*_Fe_ and *N*_O_ are the numbers of iron and oxygen atoms, respectively, in the respective slab; *μ*_Fe_2_O_3__ is the chemical potential of the respective bulk Fe_2_O_3_ per formula unit; *μ*_O_ is the chemical potential of oxygen vapour and taken as half of the chemical potential of oxygen molecule and *A* is the surface area in each slab. Due to the non-centrosymmetric nature of the ε-Fe_2_O_3_ bulk structure, the calculation of the surface energy of bottom surface is done with the help of a symmetric slab of ε-Fe_2_O_3_ (as explained in our previous work^27^) which has the same top surface as that of ε-Fe_2_O_3_ slab considered here.

For all energies in eqn (1) and (2) we employ the DFT total energies, neglecting entropic effects, which however will not affect the relative stability of the interface.^62^

The interface energy calculated for the heterostructure with ferromagnetic and anti-ferromagnetic coupling at the interface between the two slabs is 0.099 and 0.086 eV Å^−2^, respectively. Comparing the two numbers we find that the magnetic coupling at the interface is slightly preferred to be anti-ferromagnetic. The calculated interface energy is positive, but very small, which is indicating that the formation of a heterostructure is energetically not hindered.

### Electronic properties and interface states

**Fig. 4:**
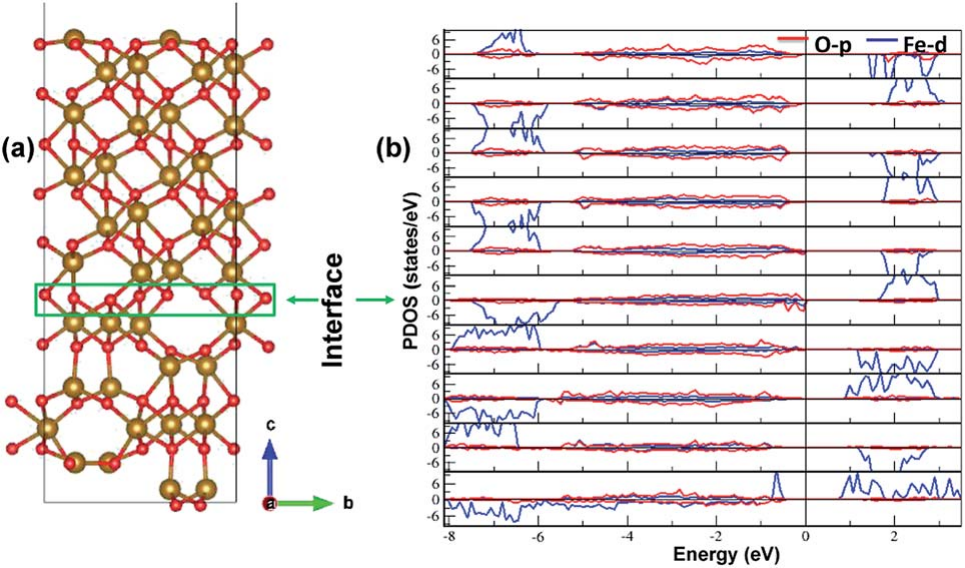
The interface of ε-Fe_2_O_3_ and α-Fe_2_O_3_ along with the partial density of states (PDOS) of the heterostructure in a layer-wise manner. Red and blue curve in the PDOS represents the density of O-p and Fe-d orbitals, respectively.

The electronic structure is shown in layer wise manner along with the heterostructure in Fig. 4. The heterostructure is divided into layers such that each partial density of states (PDOS) correspond to each layer consisting of two formula units of Fe_2_O_3_ except for the first-bottom and the tenth-top PDOS. The first-bottom PDOS consists of two formula units of Fe_2_O_2_ and the top tenth-layer PDOS consists of Fe_2_O_6_.

**Fig. 5:**
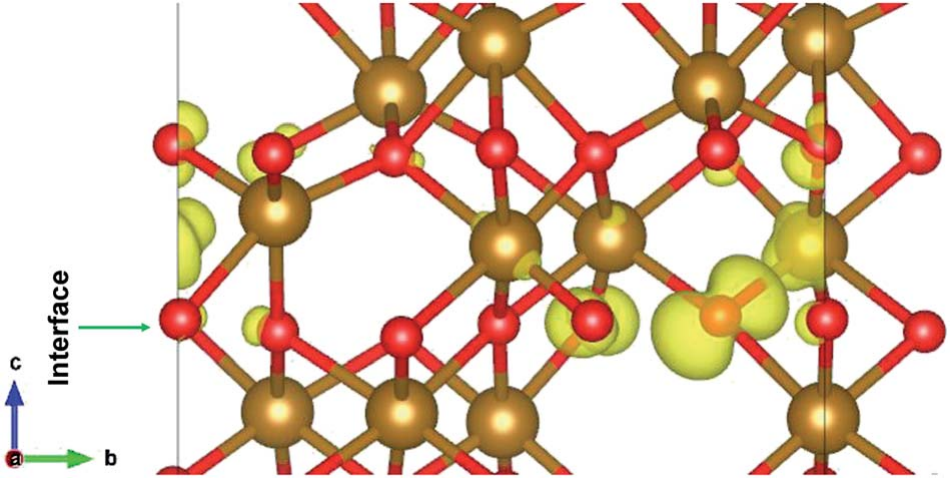
The interface of ε-Fe_2_O_3_ and α-Fe_2_O_3_ showing the orbitals contributing to the highest occupied states at the interface.

The interface formed (marked as a green rectangle in Fig. 4(a)) between the two slabs of Fe_2_O_3_ polymorphs is made up of O-atoms of ε-Fe_2_O_3_. As it is seen from the partial density of states (PDOS) in Fig. 4(b), each layer of the heterostructure is an insulator and no conducting state appears at the interface. A sharp peak below the Fermi level in the first bottom layer of PDOS corresponds to a surface state due to the d-orbitals of Fe-atoms. In the interface layer, fifth from the bottom, there are states appearing just below the Fermi level which are otherwise not present in any of the layers. These states correspond to the highest occupied molecular orbital (HOMO) and are mainly contributed from the O-atoms in the interface. This is also evident from the partial charge density corresponding to the HOMO as shown in Fig. 5. The Fe-atoms in the interface layer of the DOS just above the O-atoms also contribute, but very weakly, in the HOMO.

### Charge-density difference

**Fig. 6:**
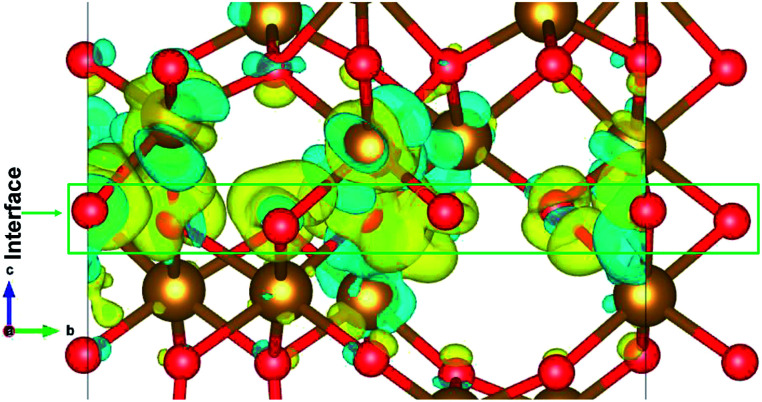
The charge density difference of ε/α-Fe_2_O_3_ interface heterostructure. The yellow region shows the charge accumulation and the cyan region shows charge depletion. The isosurface value is ±0.005 e Å^−3^.

The charge distribution in the heterostructure due to the formation of interface is analysed by taking the charge density difference of the heterostructure and each part of Fe_2_O_3_ slabs. The charge density difference is calculated by the use of following equation:

3 Δ*ρ* = *ρ*_ε/α_ − *ρ*_ε_ − *ρ*_α_

where *ρ*_ε/α_ is the charge density of the ε/α-Fe_2_O_3_ heterostructure, *ρ*_ε_ is the charge density of the ε-Fe_2_O_3_ part of the heterostructure and *ρ*_α_ is the charge density of the α-Fe_2_O_3_ part of the heterostructure. The charge density difference is shown in Fig. 6.

The charge density difference shows that the charge is redistributed mainly at the interface of ε- and α-Fe_2_O_3_. The maximum charge accumulation is at the interface, on the O-atoms which belong to ε-Fe_2_O_3_ and also very small on Fe-atoms above the interface. The Fe-atoms above the interface belonging to α-Fe_2_O_3_ have more charge depletion. The Fe-atoms below the interface belonging to the ε-Fe_2_O_3_ side have very little or no charge accumulations. This charge redistribution suggests that the electron is transferred from α-Fe_2_O_3_ to the ε-Fe_2_O_3_ slab and the holes remains on the bottom Fe-atoms of the α-Fe_2_O_3_ slab. The localization of charges on the atoms at the interface does not contributes to any conducting states, making the heterostructure act like an insulator. The transfer of charges form one material to the other leads to net charge accumulation and thus creates a built-in electric field at the interface. This electric field can contribute to a more efficient separation of electrons and holes in the heterostructure, thus suppressing charge recombination.^63^

### Electrostatic potential and the band offsets

**Fig. 7:**
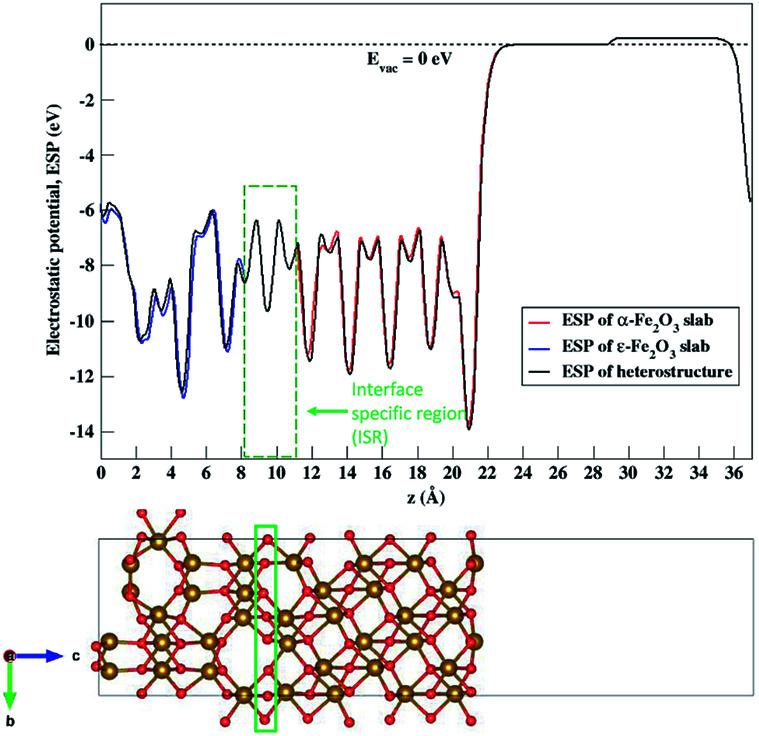
Average electrostatic potential (ESP) of the separate slabs of ε-Fe_2_O_3_ (blue curve), α-Fe_2_O_3_ (red curve) and their complete heterostructure (black curve). The broken line represents the vacuum potential. The rectangle in green broken lines represents the interface specific region (ISR).

The nature of the electronic energy levels plays an important role for the use of materials in energy applications. The formation of a heterostructure interface of two semiconductors requires that their vacuum levels align at the interface. This is known as Anderson's electron affinity rule.^64,65^ The band positions with respect to the vacuum energy levels were obtained from our DFT calculations on the optimized slabs of ε-Fe_2_O_3_ and α-Fe_2_O_3_ separately. Since the separate slabs of ε- and α-Fe_2_O_3_ are not dipole neutral, a dipole correction was applied for obtaining the correct value of vacuum potential. With the correct value of the vacuum potential, the average electrostatic potential (ESP) of the slab of ε-Fe_2_O_3_, α-Fe_2_O_3_ and their complete heterostructures with a common reference vacuum level is plotted and shown in Fig. 7.

**Fig. 8:**
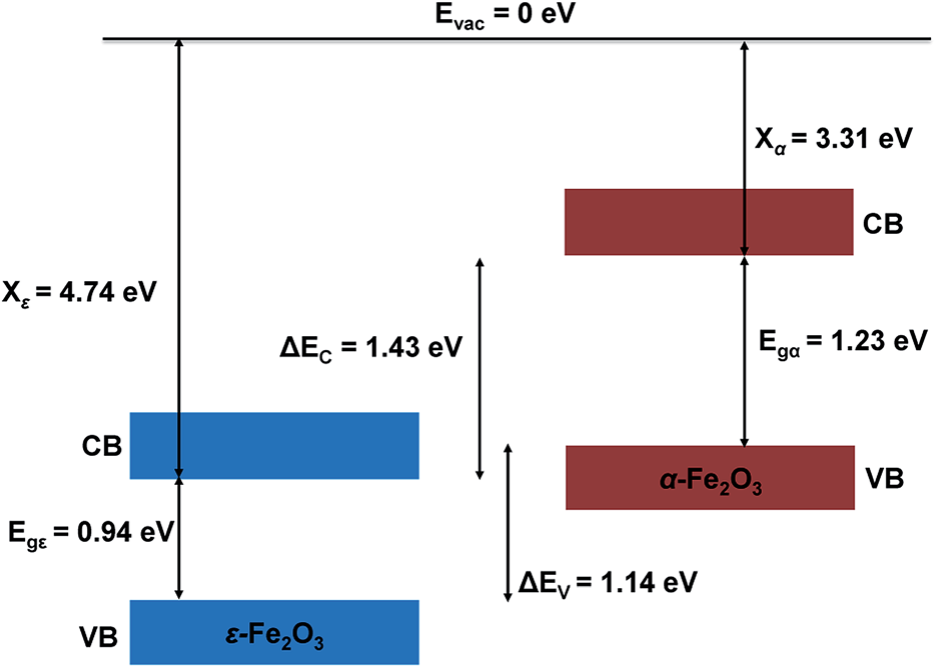
Schematic diagram showing the band alignment of ε-Fe_2_O_3_ and α-Fe_2_O_3_ slabs before connection.

The ESP of the heterostructure matches well with the ESP of the individual separate slabs of ε- and α-Fe_2_O_3_. The O-atoms of ε-Fe_2_O_3_ slabs contributes in interface formation, so they are counted as the interface and lie in the interface specific region (ISR). The position of the valence band and conduction band with respect to the vacuum energy level was calculated for both the separate iron-oxide slabs and plotted as shown in Fig. 8. The conduction band CB, valence band VB, electron affinity *χ* and band gap *E*_g_ are shown for both the slabs in Fig. 8. The subscript ε and α in the band gap and electron affinity represents that these quantities are associated with the slab of ε- and α-Fe_2_O_3_, respectively.

**Fig. 9:**
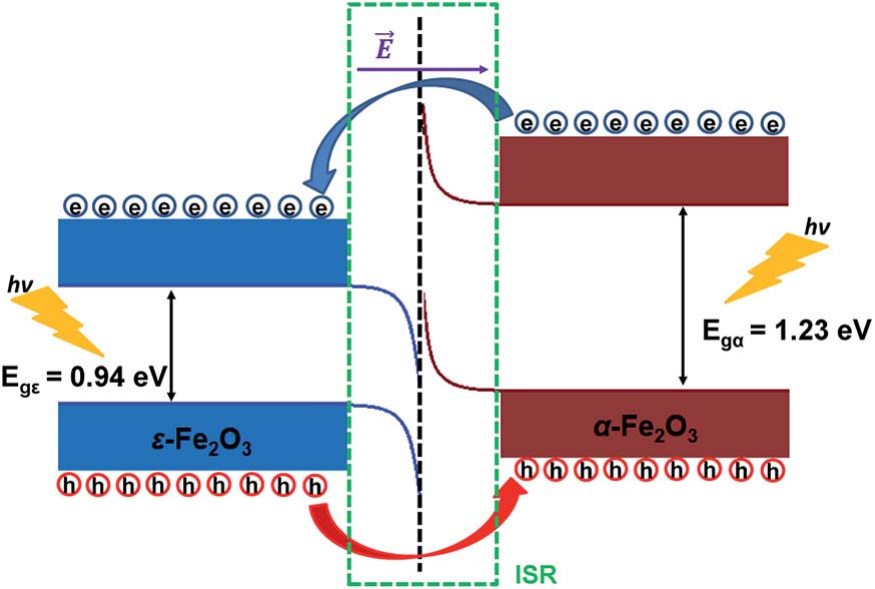
Schematic diagram showing the band bending and the charge flow at the interface of ε-Fe_2_O_3_ and α-Fe_2_O_3_ heterostructure after connection.

From Fig. 8 it is clear that the valence band edge of α-Fe_2_O_3_ is higher than the conduction band edge of ε-Fe_2_O_3_. Both band edges of α-Fe_2_O_3_ are higher than that of ε-Fe_2_O_3_ and this arrangement of band edges falls in the category of type-III broken-gap heterostructures.^66^ Since the band edges of α-Fe_2_O_3_ lies above the band edges of ε-Fe_2_O_3_, the work function of α-Fe_2_O_3_ will be lower than that of the ε-Fe_2_O_3_. Before the formation of the interface in the heterojunction, the band of each slab system is unaffected by each other. As soon as the junction is formed and the charges flow spontaneously across it and reach equilibrium, a band bending occurs at the interface. This flow of charges takes place across the interface and a built in voltage is developed across it. The direction of the electric field across the interface will be from ε- to the α-Fe_2_O_3_ as shown in Fig. 9.

The band bending at the interface of the heterostructure is shown in Fig. 9. When the photons are incident on the heterostructure, the generation of electron–hole pairs takes place and the electron jumps to the CBs in both the Fe_2_O_3_ slabs leaving the holes in the VBs of the respective Fe_2_O_3_ material. Due to the nature of the alignment of the bands and the difference in their respective conduction band edges, which is the conduction band offset (CBO), the electrons flow from the CB of α-Fe_2_O_3_ to the CB of ε-Fe_2_O_3_. Similarly, the holes flow from the VB of ε-Fe_2_O_3_ to the VB of α-Fe_2_O_3_. This type-III of band alignment at the interface results in the separation of photogenerated charges. The electron prefers to reside in ε-Fe_2_O_3_ and the hole prefers α-Fe_2_O_3_.

Since the valence band maximum (VBM) of the α-Fe_2_O_3_ slab is higher than the conduction band minimum (CBM) of ε-Fe_2_O_3_, the electron can tunnel from the VBM of α-Fe_2_O_3_ to the CBM of ε-Fe_2_O_3_. This band-to-band tunneling (BTBT) in type-III heterostructure results in negative differential resistance (NDR) and can be used in tunnel field-effect transistors (TEFT).^67–70^

## Conclusion

In summary, we have modelled the heterostructure of the two readily available polymorphs of Fe_2_O_3_. Both ε-Fe_2_O_3_ and α-Fe_2_O_3_ are charge-transfer insulators and their heterostructure also remains an insulator. The interface energy explains the anti-ferromagnetic spin arrangement at the interface which results in overall reduced magnetization. There is a localization of charges at the interface which occurs because of the strain at the interface between the two slabs of Fe_2_O_3_. The charge density difference also suggests that electrons are localized at the interface on oxygen atoms of ε-Fe_2_O_3_ and holes above the interface on iron atoms of α-Fe_2_O_3_. The band alignment with respect to a reference vacuum potential at zero eV, gives a rare type-III heterostructure. The band bending at the interface shows the transfer of electrons from α-Fe_2_O_3_ to the ε-Fe_2_O_3_ and the holes from ε-Fe_2_O_3_ to α-Fe_2_O_3_. The heterostructure showing charge separation at the interface reduces the recombination rate of the photo-generated electron–hole pairs and can thus give better efficiency in comparison to the use of a single material as photoelectrode in PEC cells. The broken band type-III heterostructure can show band-to-band tunneling and find applications in field-effect transistors.

## Conflicts of interest

There are no conflicts of interest to declare.

## Supplementary Material
